# *Aspergillus* Section *Fumigati* in Firefighter Headquarters

**DOI:** 10.3390/microorganisms9102112

**Published:** 2021-10-07

**Authors:** Carla Viegas, Bianca Gomes, Marta Dias, Elisabete Carolino, Liliana Aranha Caetano

**Affiliations:** 1 H&TRC—Health & Technology Research Center, ESTeSL—Escola Superior de Tecnologia da Saúde de Lisboa, Instituto Politécnico de Lisboa, 1990-096 Lisbon, Portugal; bianca.gomes@estesl.ipl.pt (B.G.); martasfd@gmail.com (M.D.); etcarolino@estesl.ipl.pt (E.C.); liliana.caetano@estesl.ipl.pt (L.A.C.); 2 Public Health Research Centre, NOVA National School of Public Health, Universidade NOVA de Lisboa, 1099-085 Lisbon, Portugal; 3 Comprehensive Health Research Center (CHRC), NOVA Medical School, Universidade NOVA de Lisboa, 1169-056 Lisbon, Portugal; 4 Research Institute for Medicines (iMed.uLisboa), Faculty of Pharmacy, University of Lisbon, 1649-003 Lisbon, Portugal

**Keywords:** sampling approach, culture-based methods, molecular tools, azole resistance profile

## Abstract

Background: *Aspergillus* section *Fumigati* is one of the *Aspergillus* sections more frequently related to respiratory symptoms and by other health outcomes. This study aimed to characterize *Aspergillus* section *Fumigati* distribution in eleven firefighter headquarters (FFHs) to obtain an accurate occupational exposure assessment. Methods: A sampling approach protocol was performed using active (impaction method) and passive sampling methods (floor surfaces swabs, electrostatic dust collectors (EDCs), and settled dust). All samples were analysed by culture-based methods and passive sampling was used for molecular detection of *Aspergillus* section *Fumigati.* Results: Of all the matrices, the highest counts of *Aspergillus* sp. were obtained on settled dust filters (3.37% malt extract agar—MEA, 19.09% dichloran glycerol—DG18) followed by cleaning cloths (1.67% MEA; 7.07% DG18). Among the *Aspergillus* genus, the *Fumigati* section was predominant in Millipore and EDC samples in MEA (79.77% and 28.57%, respectively), and in swabs and settled dust filters in DG18 (44.76% and 30%, respectively). The *Fumigati* section was detected more frequently in DG18 (33.01%) compared to MEA (0.33%). The *Fumigati* section was observed in azole supplemented media (itraconazole and voriconazole) in several passive sampling methods employed and detected by qPCR in almost all passive samples, with EDCs being the matrix with the highest prevalence (*n* = 61; 67.8%). Conclusion: This study confirms that *Aspergillus* sp. is widespread and the *Fumigati* section is present in all FFHs. The presence of fungi potentially resistant to azoles in the FFHs was also observed. Further studies are needed to identify the best corrective and preventive measures to avoid this section contamination in this specific occupational environment.

## 1. Introduction

*Aspergillus* species are filamentous fungi commonly observed in different environmental compartments such as soil, water and air, with an emphasis on decaying vegetation, seeds and grains, where they prosper as saprophytes. *Aspergillus* species are also found in different indoor environments, and some species are considered opportunistic pathogens for humans [1,2]. *Aspergillus* conidia can be abundant in outdoor and indoor environments and are easily dispersed in the air depending on the developed activities. Since the conidia are very small, they are easily inhaled and may colonize the upper and lower respiratory tract of exposed individuals [2,3,4]. *Aspergillus* section *Fumigati* is one of the *Aspergillus* sections more frequently related to respiratory symptoms due to the small size of the conidia, thermotolerance, its nutritional versatility, and several other virulence factors [2,5,6,7]. Additionally, the development of resistance to antifungal drugs, mainly in this *Aspergillus* section, is a phenomenon with growing prevalence in Europe, being associated with therapeutic failure and high mortality rates [8].

An approach to ensure an accurate *Aspergillus* section *Fumigati* exposure assessment, considering its clinical and toxicological relevance, was recently suggested for occupational environments [4]. Among the recommendations, the combination of active and passive sampling methods, the use of culture-dependent and -independent methods, and the screening of azole resistance were emphasized and extensively justified by the results of previous assessments [4,7,9,10,11,12,13,14,15,16].

Several occupational environments in Portugal have already been characterized regarding the *Aspergillus* genus prevalence, demonstrating its critical dissemination indoors [4,7,9]. More recently, an assessment of Portuguese firefighters’ ambulances identified hazardous levels of *Aspergillus* section *Fumigati* in ambulance air, which would be able to reach the alveoli [17,18]; additionally, there are other relevant findings, such as toxigenic fungi with clinical relevance found in ambulance air, contamination of surfaces increased after cleaning at some sites and mycotoxins detected in mops and electrostatic dust cloths [18]. Thus, a concern regarding microbiological contamination in the headquarters was raised and the requirement for a profound characterization was identified. This study aimed to characterize *Aspergillus* section *Fumigati* distribution in firefighter headquarters following previous recommendations [4] to obtain an accurate occupational exposure assessment.

## 2. Material and Methods

### 2.1. Firefighter Headquarters Characterization

**Figure 1:**
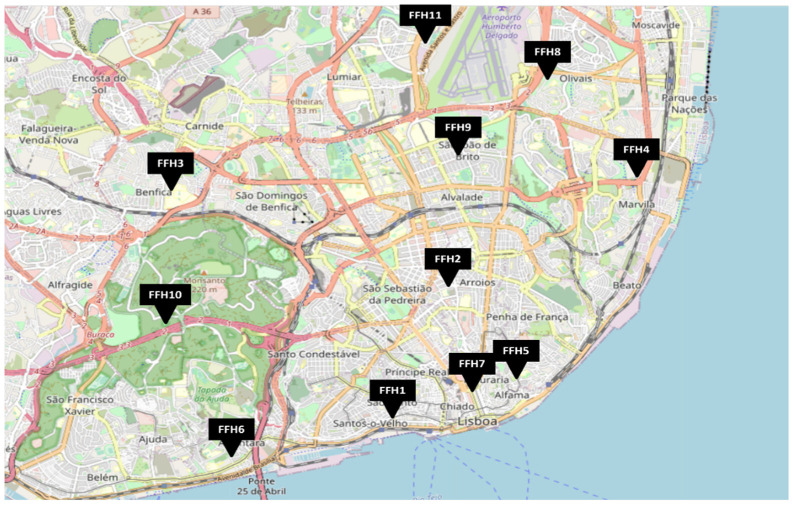
Geographical distribution of the FFH (Firefighter headquarter) assessed.

This study is part of an enlarged exploratory study aiming to assess microbial contamination in firefighter headquarters (FFHs). Eleven FFHs located in the Lisbon area were assessed between September 2020 and May of 2021 (Figure 1).

In general, all FFHs were composed by dormitory, balneary, kitchen, canteen, bar, living room, administrative room, reception and gym, these being the chosen places for the sampling campaign. A local characterization of each FFH and the corresponding sampling sites was performed before all the procedures. The sampling campaign covered a range of facilities types, from centenary buildings to more recent ones, the latter being until two years old. The number of occupants per turn ranged from seven to fifty. Visible problems in wall conditions such as cracks, infiltrations, air leaks and mould growth were also registered, detected in older FFHs. Regarding the cleaning routine, all areas of the headquarters were cleaned and disinfected once a day (Table 1).

### 2.2. Sampling Approaches

**Figure 2:**
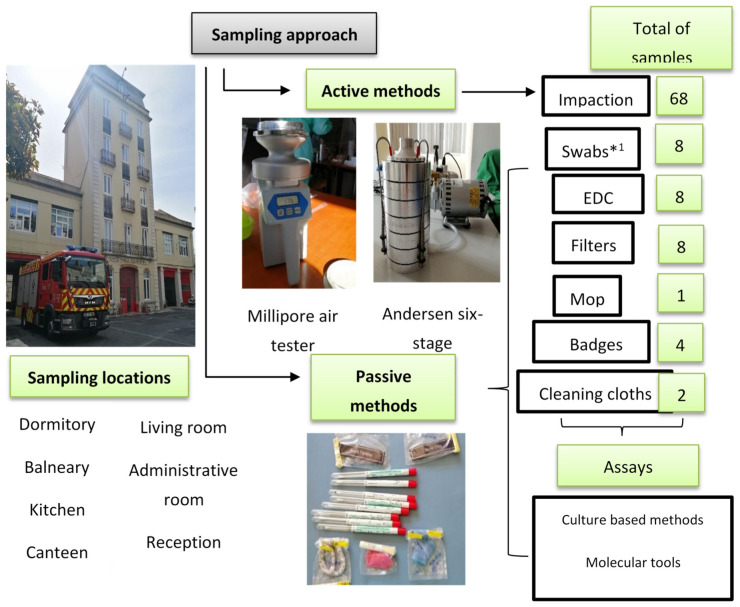
Sampling approach and assays applied in FFH2 assessment; EDC: Electrostatic dust collector. *^1^ swabs were analysed only through culture-based methods.

At each chosen site, a sampling approach protocol was performed using active and passive sampling methods (Figure 2). Indoor air was collected from selected areas by the impaction method on each plate using two devices (Millipore air tester and Andersen six-stage air sampler), according to the manufacturers’ guidelines. Different culture media were used to promote selectivity of bacteria and fungi [18,19].

The following passive sampling methods were used: floor surfaces swabs, electrostatic dust collectors (EDCs), and settled dust. For the collection of settled dust, a vacuum cleaner equipped with a filter (for further analysis) was used and a composite sample [20] of the settled dust from each FFH was obtained. Cloths and mops used in cleaning routines were collected, as well as the identification badges from firefighters’ uniforms.

A total of 760 indoor air samples were collected by the impactors, 190 samples from each culture medium. Regarding passive sampling methods, 82 EDCs, 90 swabs, 90 filters, 11 settled dust composite samples, 67 firefighter uniform badges, 25 cleaning cloths and 14 mops were obtained. Table S1 presented the sample distribution per sampling method in each FFH.

Indoor air was collected through active sampling methods in each selected area. About 250 L were collected at a flow rate of 140 L/min by Millipore air tester (Millipore, Billerica, MA, USA). A six-stage Andersen air sampler was also used, with a flow rate of 28.3 L/min, for 9 min in each culture medium, according to manufacturer’s recommendations [18]. An outdoor air sample form each FFH was taken for reference.

Concerning passive sampling methods, the floor surfaces were swabbed using a 10 × 10 cm square stencil, which was disinfected between each sample with a 70% alcohol solution [11]. The EDCs were placed in each sampling area 1.5 m above the ground for 30 days [21]. All samples (filters, settled dust, mops, cleaning cloths and identification badges) were kept in sterilized bags and transported under refrigeration (0–4 °C) to the laboratory [18].

Swabs were washed with 1 mL of 0.1% Tween 80 saline (0.9% NaCl) for 30 min on the orbital shaker (250 rpm, 30 min). The identification badges and a piece (2 cm^2^) of each settled dust filter, cleaning cloth and mop were processed similarly with 10 mL of the same solution [12]. EDCs were weighted and processed with 20 mL of the same washing solution. A composite sample with the settled dust obtained from each FFH was washed in a ratio of 1 g per 9.1 mL of 0.1% Tween 80 saline (0.9% NaCl) for 30 min at 250 rpm [18].

### 2.3. Aspergillus Section Fumigati Prevalence

All samples were analysed by culture-based methods. The impacted air and 150 µL of the suspensions resulting from washing the passive samples were inoculated in two different culture media: malt extract agar (MEA) supplemented with chloramphenicol (0.05%) (Frilabo, Maia, Portugal) and dichloran glycerol (DG18) agar supplemented with chloramphenicol (0.01%) (Frilabo, Maia, Portugal). The same extracts were used to screen for azole resistance by inoculating 150 µL of the samples onto Sabouraud dextrose agar (SDA) supplemented with 4 mg/L itraconazole (ITR), 2 mg/L voriconazole (VOR), 0.5 mg/L Posaconazole (POS), or SDA alone (as control) adapted from the [22]. The reference strain *A. fumigatus* ATCC 204305 was used as negative control and a pan-azole-resistant strain was used as positive control (both kindly provided by Reference Unit for Parasitic and Fungal Infections, Department of Infectious Diseases of the National Institute of Health, from Dr. Ricardo Jorge).

All plates, including those of the air impaction devices used and those where the extracts of the passive samples were inoculated, were incubated at 27 °C and examined for *Aspergillus* sp. densities (colony-forming units, CFU·m^−3^, CFU·m^−2^, CFU·g^−1^) after 3 (azole-resistance screening) and 5–7 days (MEA and DG18). This temperature was selected to enable the identification of all fungi in samples, for a broader characterization of fungal contamination, besides *Aspergillus* section *Fumigati*.

Fungal densities were determined depending on the passive sampling method used and applying appropriate formulas (Table S2) as previously described [9,10] Fungal isolates were identified by macroscopic and microscopic morphology using tease mount or Scotch tape mount and lactophenol cotton blue mount procedures [23]. Whenever colony overgrowth was observed due to fungi with fast growing rates (*Chrysonilia sitophila*, *Trichoderma* sp. and *Mucorales* order), a quantitative cut off of 500 isolates (CFU) was applied for air sampling and the mean of the results obtained from each environmental matrix was used for passive sampling [9,10,12,13].

### 2.4. Molecular Detection of Aspergillus Section Fumigati

Samples extracts (8.8 mL) from passive sampling (excluding surface swabs) were used for molecular detection of *Aspergillus* section *Fumigati* (Table 2). Fungal DNA was extracted using the ZR Fungal/Bacterial DNA MiniPrep Kit (Zymo Research, Irvine, USA) and molecular identification was performed by real-time PCR (qPCR) using the CFX-Connect PCR System (Bio-Rad). Reactions included 1× iQ Supermix (Bio-Rad, Portugal), 0.5 μM of each primer, and 0.375 μM of TaqMan probe in a total volume of 20 μL. Amplification followed a three-step PCR: 40 cycles with denaturation at 95 °C for 30 s, annealing at 52 °C for 30 s, and extension at 72 °C for 30 s.

A non-template control and a positive control consisting of DNA obtained from a reference that belonged to the culture collection of the Reference Unit for Parasitic and Fungal Infections, Department of Infectious Diseases of the National Institute of Health, from Dr. Ricardo Jorge. These strains have been sequenced for ITS, B-tubulin, and Calmodulin.

### 2.5. Statistical Analysis

Data were analysed using SPSS statistical software, V26.0, for Windows (Microsoft, USA). The results were considered significant at the 5% significance level. To test the normality of the data, the Shapiro–Wilk test (n’s < 50) or the Kolmogorov–Smirnov test (n’s > 50) was used. To compare Fungal contamination, *Aspergillus* sp. and *Aspergillus* section *Fumigati* between FFHs, media, sampling method and sections, the Kruskal–Wallis test was used, since the assumption of normality was not verified.

To study the relationship between fungal contamination, *Aspergillus* sp. and *Aspergillus* section *Fumigati*, Spearman’s correlation coefficient was used, since the normality assumption was not verified. To study the relationship between sections, FFHs, media and sampling method, the Chi-Square test by Monte Carlo simulation was used, since the assumptions of applicability of the Chi-Square test were not verified. To identify the association trend, multiple correspondence analysis was used, having been discretized the variables fungal contamination (<53.76, [53.76; 294.2[, [294.2; 2052.05[, ≥2052.05), *Aspergillus* sp. (<3.93, [3.93; 100[, ≥100) and *Aspergillus* section *Fumigati* (<3.93, [3.93; 11.78[, [11.78; 212.31[, ≥212.31), considering the quartiles.

## 3. Results

### 3.1. Aspergillus Section Fumigati Distribution

Among all the FFHs, and concerning *Aspergillus* genera, the highest value obtained by the Andersen six-stage air samples was observed on FFH5 (3.81%), and the same trend was obtained from the cleaning cloths and filters from the same FFH (0.51% and 7.33%). Concerning Millipore air samples, the FFH10 presented the highest counts (1.87%). *Fumigati* was the predominant section in Andersen six-stage FFH7 (88.37%), while for Millipore air samples, the highest counts were observed in FFH9 (100%).

In FFH4, the *Aspergillus* genera was predominant in EDCs (0.55%) and in settled dust (0.65%) samples, while in FFH2, the section *Fumigati* was the most frequent in EDCs (100%). *Aspergillus* sp. was identified in mops from FFH1 (0.44%), *Fumigati* being the predominant section (100%). Similar results were obtained in swabs samples from FFH3, *Aspergillus* being the most frequent genera (0.08%) and *Fumigati* being the prevalent section (100%).

Regarding samples collected from all FFHs, the genus *Aspergillus* was present in almost all matrices, with a prevalence of 1.52% in MEA (Millipore; six-stage Andersen; EDCs; cleaning cloths; mops; settled dust filters; swabs) and 2.20% in DG18 (Millipore; six-stage Andersen; EDCs; cleaning cloths; settled dust filters; swabs), being absent in mops, identification badges and settled dust samples in MEA and identification badges in DG18 (Table 3).

**Figure 3:**
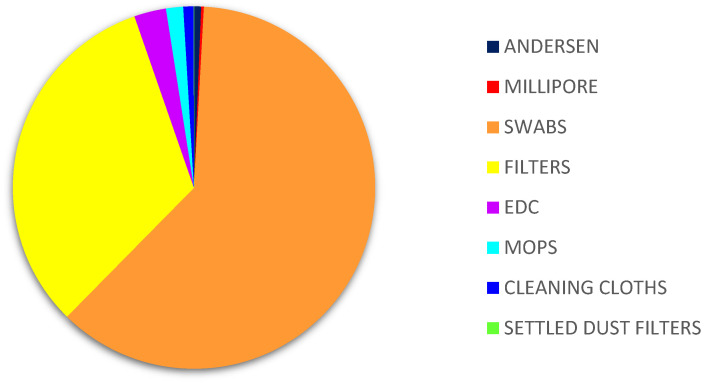
*Aspergillus* sp. distribution in samples on DG18. DG18: Dichloran–Glycerol Agar; EDC: Electrostatic dust collector.

Of all the matrices, the highest counts of *Aspergillus* sp. were obtained on filters (3.37% MEA, 19.09% DG18) followed by cleaning cloths (1.67% MEA; 7.07% DG18). The matrices where the lowest prevalence was reported were air samples from Millipore (0.10% MEA, 0.77% DG18), six-stage Andersen (0.11% MEA) and settled dust (0.89% DG18) (Figure 3).

**Figure 4:**
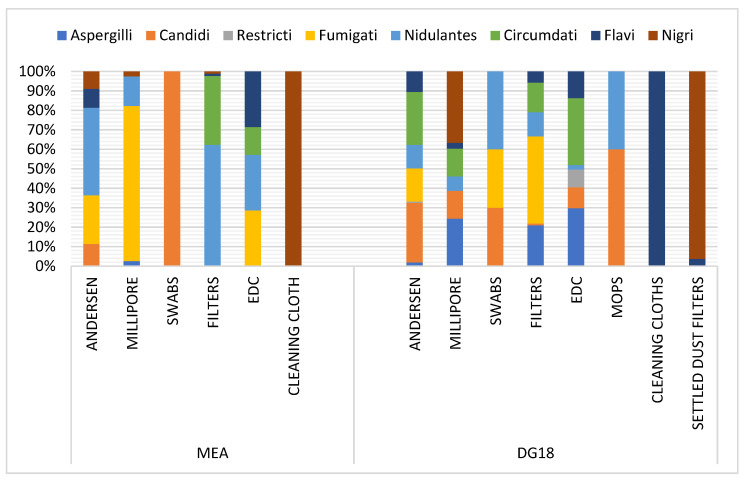
Distribution of *Aspergillus* sections per matrice in MEA (Malt Extract Agar) and DG18 (Dichloran–Glycerol Agar).

Among the *Aspergillus* genus, the *Fumigati* section was predominant in Millipore and EDC samples in MEA (79.77% and 28.57%, respectively), and in swabs and settled dust filters in DG18 (44.76% and 30%, respectively). This section was also observed in six-stage Andersen samples (25.04% MEA; 16.99% DG18) (Figure 4). Among *Aspergillus* sp., section *Fumigati* was identified in the Andersen air sampler through all six stages. In FFH8, the section was the only one identified (100% on DG18) in stage 1 (7 µm). In FFH2, despite the lower frequency (4% MEA), the section was reported on the 2nd stage (4.7 µm). The section was the only one found on DG18 in stage 3 (3.1 µm) in FFH7. The same trend was obtained in FFH2 (100% MEA) in stage 4 (2.1 µm) and in FFH6 (100% MEA) in stage 5 (1.1 µm). In addition, FFH5, FFH6 and FFH10 had similar results (100% MEA) in stage 6 (0.65 µm). In stage 6, section *Fumigati* was the only found in the two culture media from FFH8 (100% MEA and DG18). Concerning the *Fumigati* section, positive samples (*n* = 23), the section was more frequently detected in samples performed by the Andersen sampling device (Table S3) and was more frequently detected in DG18 (33.01%) compared to MEA (0.33%) (Table S4).

### 3.2. Screening of Azole Resistance

Passive matrices (82 EDC, 102 swabs, 89 filters, 11 settled dust, 67 uniform name tags, 25 cleaning cloths and 14 mops) were extracted as described and screened for antifungal resistance to three commonly used medical azoles. Growth of *Aspergillus* sp. was observed in Sabouraud (SDA: 1.11%) and in two azole-supplemented SDA media (ITR: 0.11%; VOR: 0.11%), with no growth observed in the POS media. The *Fumigati* section was the only among *Aspergillus* sp. observed in three culture media (SDA: 8.9%; ITR: 100%; VOR: 85.3%) (Table 4).

The relative frequency of azole-resistant *Aspergillus* section *Fumigati* isolates among all *Aspergillus* sp. isolates in the azole resistance screening was 17.8%. Isolates able to grow in 4 mg/L itraconazole and/or 2 mg/L voriconazole (all of them from *Aspergillus* genus) were identified in three different headquarters, from the following samples: 2 filters in FFH1; 1 EDC in FFH3; and 4 EDCs in FFH6.

Of all screened matrices, *Aspergillus* sp. was identified only in mops, EDCs, settled dust filters and settled dust. Concerning the *Aspergillus* sections, the *Fumigati* section was prevalent in the SDA of 100% in mops, 4.4% in EDCs and 3.7% in filters. *Fumigati* was the only section found in ITR (filters and EDCs: 100%) and the most predominant in VOR (EDCs: 97.1%) (Table 4).

**Figure 5:**
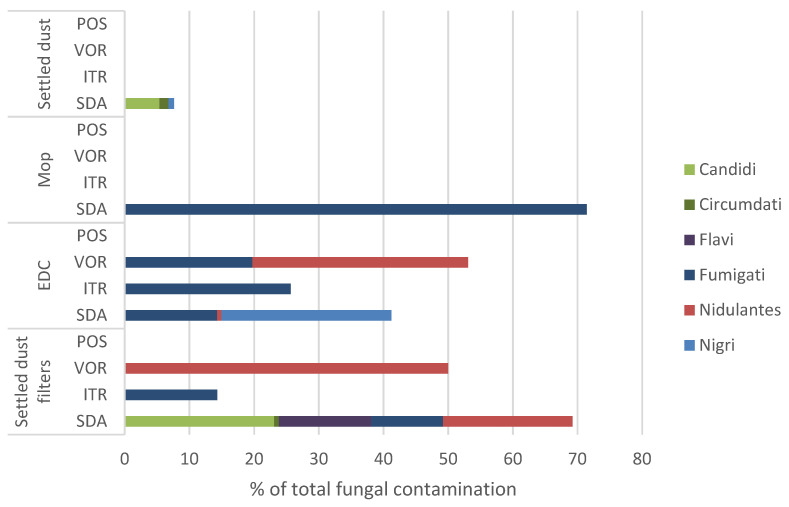
Relative distribution of *Aspergillus* sections per matrix type in SDA and azole-supplemented SDA media (ITR, VOR, POS) regarding total fungal contamination. SDA (Sabouraud Dextrose Agar); ITR (Itraconazole); VOR (Voriconazole); POS (Posaconazole); EDC (Electrostatic dust collector).

The results of *Aspergillus* sp. relative distribution per sample type and culture media are depicted in Figure 5. The *Fumigati* section was predominant among *Aspergillus* sp. in mop samples in SDA media, abundant in EDCs and settled dust filter samples, including in ITR and VOR, and absent in settled dust.

### 3.3. Molecular Detection

The *Aspergillus* section *Fumigati* was detected by qPCR in almost all passive samples, with EDCs being the matrix with the highest prevalence (*n* = 61; 67.8%) followed by settled dust filters (*n* = 60; 66.6%). This section was also detected in seven mops (50%), in 15 cleaning cloths (60%), and, to a lesser extent, in identification badges (*n* = 4; 5.9%) (Table S5).

### 3.4. Correlation Analysis

Regarding the comparison of fungal contamination, *Aspergillus* sp. and *Aspergillus* section *Fumigati* between sampling methods, statistically significant differences were detected in (i) fungal contamination ($\chi_{K-W}^{2}\left(5\right)=115.897$, *p* = 0.000); (ii) *Aspergillus* sp. ($\chi_{K-W}^{2}\left(5\right)=149.849$, *p* = 0.000). In both cases, it was found that settled dust filters, EDCs and the group of others (mops, swabs and cleaning cloths) were the sampling methods that presented the highest values (Table 5).

Among the media used (MEA, DG18, SDA, ITR, VOR, and POS), statistically significant differences were detected for fungal contamination ($\chi_{K-W}^{2}\left(3\right)=20.273$, *p* = 0.000), *Aspergillus* sp. ($\chi_{K-W}^{2}\left(3\right)=27.499$, *p* = 0.000) and *Aspergillus* section *Fumigati* ($\chi_{K-W}^{2}\left(3\right)=39.411$, *p* = 0.000). It was found that the SDA and ITR+VOR were the ones with the highest values (Table 6).

Regarding FFHs, statistically significant differences were detected for fungal contamination ($\chi_{K-W}^{2}\left(10\right)=22.200$, *p* = 0.014), *Aspergillus* sp. ($\chi_{K-W}^{2}\left(10\right)=22.227$, *p* = 0.014) and *Aspergillus* section *Fumigati* ($\chi_{K-W}^{2}\left(10\right)=22.481$, *p* = 0.013). Regarding fungal contamination, FFHs 3, 7, 8, 9 and 11, were the ones with the highest values, while for *Aspergillus* sp., FFHs 3, 6, 7 and 9 were the ones presenting the highest values. Concerning to the *Aspergillus* section *Fumigati*, FFHs 6, 7 and 8, were the ones where the highest values were observed (Table 7).

Significant correlations were detected between *Aspergillus* sp. and *Aspergillus* section *Fumigati* (rS = 0.137, *p* = 0.036) and with fungal contamination (rS = 0.705, *p* = 0.000) and between *Aspergillus* section *Fumigati* and fungal contamination (rS = 0.130), *p* = 0.047). These results reveal that higher values for *Aspergillus* sp. are related to higher counts for *Aspergillus* section *Fumigati* and fungal contamination, as well as between *Aspergillus* section *Fumigati* and fungal contamination (Table 8).

**Figure 6:**
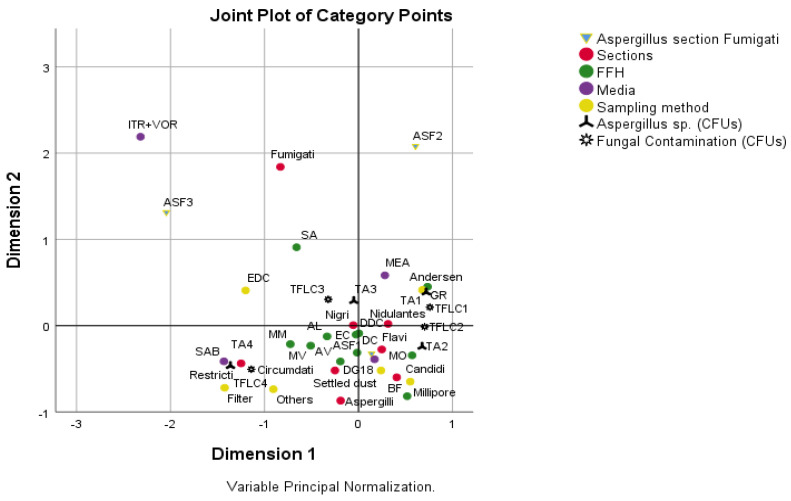
Results of multiple correspondence analysis. Study of the association between *Aspergillus* section *Fumigati*, sections, FFHs, media, sampling method, *Aspergillus* sp. and fungal contamination. *Aspergillus* section *Fumigati*: ASF1—[0; 3.93[, ASF2—[3.93; 100[, ASF3—≥100; *Aspergillus* sp. (CFUs—Colony forming units;): TA1—<3.93, TA2—[3.93; 11.78[, TA3—[11.78; 21.31[, TA4—≥21.31; Fungal contamination (CFUs): TFLC1—<53.76, TFLC2—[53.76; 294.2[, TFLC3—[294.2; 2052.05[, TFLC4—≥2052.05; FFH (firefighter headquarter).

Significant associations were detected between sections, FFHs, media and sampling method (p’s < 0.05, Qui-Square test by Monte Carlo Simulation). From an analysis of Figure 6, the following associations were identified: (i) FFHs 1, 3, 7, 8 and 9 with SAB, settled dust filters and other sampling methods, *Aspergillus* sp. with values ≥21.31, fungal contamination with values ≥2052.05, and sections *Restricti, Circumdati* and *Aspergilli*; (ii) FFHs 1, 2, 4, 8 and 10 with DG18, section *Fumigati* with values [0; 3.93[, *Nigri, Nidulantes, Flavi* and *Candidi* sections, Settled dust and Millipore sampling method, *Aspergillus* sp. with values [3.93; 11.78[ and fungal contamination with values [53.76; 294.2[; (iii) FFH5 with *Aspergillus* sp. with values <3.93, fungal contamination with values <53.76, MEA, *Nidulantes* section, Andersen sampling method; iv) FFH6 with EDCs, Fungal contamination with values [294.2; 2052.05[, *Aspergillus* sp. with values [11.78; 21.31[ and *Nigri* section.

## 4. Discussion

The presence of fungi in an indoor environment is influenced by a wide range of variables, such as human occupancy and their activities, humidity levels, ventilation, environmental characteristics, water infiltrations, building and decoration materials and outdoor air [25,26]. Furthermore, there is strong scientific evidence corroborating the relationship between the building dampness, visible mould, and moisture damage with adverse respiratory health effects [4,27,28,29,30,31,32]. Moisture, nutrients and temperature are proved to be the most important variables that influence the growth and dissemination of fungi on building materials [33]. Thus, the results regarding *Aspergillus* sp. and *Fumigati* section contamination in the assessed FFHs were the expected considering the observed FFH conditions (in 8 from the 11 FFHs): leakages, visible mould growth and cracks on the walls/floor.

The identification of the *Aspergillus* section *Fumigati* through passive and active sampling has already been reported [4,34,35,36,37,38]. However, other studies developed in Portuguese occupational environments presented a lower prevalence of *Aspergillus* sp. and, more specifically, section *Fumigati* [9,10,12,13,35]. Indeed, in primary health care centres the *Fumigati* section presence was 33.3% on surface swabs and 1.3% in EDCs [9,10]. In one central hospital, *Aspergillus* sp. presented an overall prevalence of 17.25%, with the *Fumigati* section only being observed in the vacuum bag [12]; in Portuguese bakeries, the *Fumigati* section was found on air samples (3.2% on DG18) and in EDCs (8.3% on MEA and 50% on DG18) [13]; in three fitness centres, only four isolates of this section were found in one air sample [35].

In this study, a multiple approach protocol was performed comprising the two sampling methods for a better characterization of contamination in FFHs. *Aspergillus* counts were revealed to be higher in settled dust filters and cleaning cloths. Previous studies also identified passive sampling as suitable to determine *Aspergillus* section *Fumigati* by culture-based methods through sampling of filtering respiratory protective devices and other environmental matrices in settings such as waste-sorting plants, veterinary clinics, dairies, ambulances, and many other indoor and occupational environments [4,10,16,18,32]. In fact, passive sampling is able to characterize contamination levels over a wider period of time, compared to air sampling [21,36]. Thus, higher fungal counts and greater fungal diversity are expected by passive sampling.

The *Aspergillus* section *Fumigati* was predominant in swabs and settled dust filters in DG18, and in EDCs in MEA, suggesting that reliable matrices for *Aspergillus* section *Fumigati* exposure assessment were chosen [4]. The significant differences in fungal counts between passive and active sampling highlight the advantages associated with a multi-approach protocol that comprises active and passive sampling simultaneously, overcoming the limitations associated with each sampling method [4,7,9,10,11,12,13,14,15,16,39].

Regardless of the lower prevalence of *Aspergillus* sp. in air samples performed by Millipore and six-stage Andersen, the *Fumigati* section was predominant in Millipore air samples in MEA. Furthermore, the underestimation of microbial contamination collected by impaction devices (Andersen six-stage and Millipore sampling devices), due to cell damage during sampling process, has already been stated [18,40,41]. However, the six-stage Andersen sampler allows the hazardous range where the *Fumigati* section has lung penetrability to be identified [17,18,41]. Indeed, in four of the assessed FFHs, section *Fumigati* at stage 6 (0.65 µm) of reaching alveoli was observed. This has the potential to cause respiratory diseases (inflammation activation) by activating macrophages, B cells and T cells [42]. The same concern was raised in a study held in Portuguese ambulances used in emergency clinical services [18].

*Aspergillus* section *Fumigati* was more frequent in DG18 compared to MEA counts. Despite the recommendation of using MEA for aerobiological studies in a Portuguese regulatory framework dedicated to the assessment of indoor air quality (IAQ) (25APA 2010), DG18 is an efficient option due to its restrictive character, inhibiting the development of fastidious fungal species [21]. The results of some fungal overgrowth on the MEA plates may have influenced the development of *Aspergillus* sp. and, more specifically, of the *Fumigati* section, due to chemical competition [43], highlighting the use of both culture media for a wider fungal characterization [4,7,9,10,11,12,13,14,15,16,39]. Sample dilution prior to inoculation (to avoid the excessive development of fast-growing fungi on media plates) was not performed due to the limitations of this option. Indeed, the removal of rare types of organisms leads to differences in species richness and diversity, decreasing competition among microorganisms, causing a probable overgrowth in some species that were not as prevalent in the original community [44].

The higher mean rank values obtained for total fungal contamination, *Aspergillus* sp. counts, and *Aspergillus* section *Fumigati* with SDA and ITR+VOR media, compared to MEA or DG18, suggest Sabouraud is a more suitable media for the recovery of fungi and *Aspergillus* sp. The use of Sabouraud as a standard medium to assess outdoor airborne fungi by air sampling was generally supported in a recent study on media comparison [45], and has also been supported in clinical applications [46]. However, Saboraud enhanced *Chrysonilia sitophila* in other performed assessments [9,18].

The ability to use protection against respiratory devices or filters as a sampling approach depends on the features of the assessed setting, activities developed and duration of use, e.g., mop sampling depends on cleaning procedures. The EDC device, on the other hand, allows for the recovery of fungal contamination in a consistent and standardized manner (regarding retention material and collection period), and it is a low-cost, low-maintenance sampling strategy that has been increasingly used in the assessment of occupational exposure to fungal burden [19] and in indoor air quality studies [21,32].

The fact that, among *Aspergillus* sections, only the *Fumigati* section was found in azole-supplemented media, confirms the presence of fungi potentially resistant to azoles in FFHs. If this azole-resistance phenotype is further confirmed by molecular analysis or antifungal susceptibility testing, it might represent a health risk for workers in this setting, especially in the FFHs where contamination by *Aspergillus* section *Fumigati* was higher. This health risk arises from the fact that azole-resistant fungi might cause invasive infections, especially in immunocompromised individuals, which are of difficult control due to the limited treatment options [2,4,8,19]. In addition to being the etiological agent of invasive aspergillosis, the *Fumigati* section is also responsible for more common respiratory symptoms such as asthma, allergic sinusitis, cough and bronchial hyperresponsiveness [47].

Culture-based methods allowed the *Aspergillus* section *Fumigati* to be identified in various matrices (settled dust filters, swabs, EDCs and air samples from Millipore and Andersen), confirming the results of molecular detection in EDCs and filters. The use of qPCR further enabled the *Fumigati* section to be detected in additional matrices (identification badges, mops and cleaning cloths) where it was undetectable by culture. This may be associated with the absence of fungal viability due to an impediment to grow in culture (e.g., due to competition for nutrients), while the molecular tools enable even non-viable microorganisms to be identified [48]. Failure to detect the *Aspergillus* section *Fumigati* by qPCR in swabs and air samples (in contrast to the results obtained by culture) may be associated with ineffective DNA extraction in sample processing, or the presence of inhibitors (such as particles from air samples), misleading the results [4,49,50]. Without diminishing the advantages of molecular analysis, classical culture-based methods are still necessary to assess the viability of pathogenic microorganisms related to their infectivity potential. Indeed, a microorganism’s viability is associated to the potential of inflammatory and cytotoxic responses and, consequently, the infection potential. Therefore, molecular tools must be used in parallel with classic methods [4,51].

Correlation was found in this study between total fungal counts, *Aspergillus* sp. counts and *Fumigati* section counts not following the trend previously found in health care environments [9]. This means that the measures used to avoid fungal contamination in this setting are also effective concerning *Aspergillus* contamination. Nevertheless, *Aspergillus* genera assessment should always be performed, as specific *Aspergillus* sections (*Flavi, Fumigati, Circumdati* and *Nidulantes*) are indicators of harmful fungal contamination when found on air samples and require intervention, as referred to by the American Industrial Hygiene Association and Portuguese regulatory framework concerning IAQ [25,52].

Thus, considering the lack of scientific information in this specific environment, further studies are needed to characterize the overall exposure to fungal contamination and other microbiological agents, as well as regarding the most suitable corrective and preventive measures used to avoid exposure. Additionally, further research on azole-resistance profile must be conducted to better estimate the risk of exposure to resistant *Aspergillus* section *Fumigati* in this setting, namely, screening azole resistance at selective conditions for *Aspergillus* section *Fumigati*, molecular analysis of resistance mutations, and antifungal susceptibility testing.

## 5. Conclusions

Overall, this study confirms the widespread nature of *Aspergillus* sp. and the presence of section *Fumigati* in all FFHs. The presence of fungi potentially resistant to azoles in FFHs was also reported. Further studies are needed to identify the best corrective and preventive measures to avoid fungal contamination in this specific occupational environment.

This study corroborates the importance of applying a wide sampling approach when assessing occupational exposure to section *Fumigati* in FFHs. The same tendency of complementarity was found between culture-based methods and molecular methods, since qPCR added the detection of the *Fumigati* section in additional matrices, while in some samples, the section was only possible to be identified and not detected by qPCR.

DG18 should be considered in future legal and technical recommendations focusing on the assessment of occupational exposure to *Aspergillus* genera.

## Figures and Tables

**Table 1:** Characterization of the 11 FFHs sampled.

Headquarter	Building Age	Number of Occupants	Cracks in the Walls /Floor	Mould Growth	Visible Problems
**FFH1** **FFH2** **FFH3** **FFH4** **FFH5** **FFH6** **FFH7** **FFH8** **FFH9** **FFH10** **FFH11**	100 * 100 * 37 20 100 * 100 * 2 100 * 62 30 4	50 12 30 11 20 16 7 16 20 20 7	Frequently Frequently Often - - - - - - - -	Frequently Frequently Often Often Frequently Often - - Often - -	Wall cracks Wall cracks Infiltrations - Infiltrations Infiltrations - - Infiltrations Infiltrations -

* Buildings more than 100 years old; FFH—Firefighter headquarter.

**Table 2:** Sequence of primers and TaqMan probes used for real-time PCR of *Aspergillus* section *Fumigati*.

Primers and Probes	Sequences	Reference
Forward Primer	5‘-CGCGTCCGGTCCTCG-3‘	
Reverse Primer	5‘-TTAGAAAAATAAAGTTGGGTGTCGG -3‘	[24]
Probe	5‘-TGTCACCTGCTCTGTAGGCCCG -3‘	

**Table 3:** *Aspergillus* sp. distribution in all matrices in MEA and DG18 from all FFHs.

Sample	MEA			DG18		
	Fungi	CFU. m^−3^/m^−2^/g	%	Fungi	CFU. m^3^ 3/m^2^/g	%
**Andersen**	Other species *Aspergillus* sp.	405,281.8 486	99.9 0.1	Other species *Aspergillus* sp.	67,915.2 1040	98.5 1.5
**Millipore**	Other species *Aspergillus* sp.	147,399.8 158.2	99.9 0.1	Other species *Aspergillus* sp	50,624.64 393	99.2 0.8
EDC *	Other species *Aspergillus* sp.	391,345 743.1	99.8 0.2	Other species *Aspergillus* sp.	210,245.9 4640.126	97.8 2.2
**Cleaning cloths**	Other species *Aspergillus* sp.	29,500 500	98.3 1.7	Other species *Aspergillus* sp.	19,700 1500	92.9 7.1
**Mops**	Other species *Aspergillus* sp.	2600 -	100 -	Other species *Aspergillus* sp.	13,500 2500	84.4 15.6
**Identification badges**	Other species *Aspergillus* sp.	45,500 -	100 -	Other species *Aspergillus* sp.	32,500 -	100 -
**Filters**	Other species *Aspergillus* sp.	3,679,700 128,500	96.6 3.4	Other species *Aspergillus* sp.	2,699,000 52,505	98.1 1.9
**Settled dust**	Other species *Aspergillus* sp.	6496.5 -	100 -	Other species *Aspergillus* sp.	2983.9 27	99.1 0.9
**Swabs**	Other species *Aspergillus* sp.	4,562,500 10,000	99.8 0.2	Other species *Aspergillus* sp.	4,131,556 100,000	97.6 2.4

* EDC (Electrostatic dust collector) results were presented in CFU.m^−2^.day^−1^; MEA—Malt Extract Agar; DG18—Dichloran–Glycerol Agar.

**Table 4:** Prevalence of *Aspergillus* section *Fumigati* among *Aspergillus* sp. in azole-supplemented SDA media.

Matrices	SDA		ITR		VOR		POS	
	CFU.m²	%	CFU.m²	%	CFU.m²	%	CFU.m²	%
**Mops**	2500	100%	-	-	-	-	-	-
EDC *	106	4.4%	1062	100%	3503	97.1%	-	-
**Settled dust filters**	1500	3.7%	1062	100%	-	-	-	-
**Total**	4106	8.9%	1562	100%	3503	85.3%	-	-

* EDC (Electrostatic dust collector) results were presented in CFU.m^−2^.day^−1.^; SDA (Sabouraud Dextrose Agar); ITR (Itraconazole); VOR (Voriconazole); POS (Posaconazole).

**Table 5:** Comparison of fungal contamination, *Aspergillus* sp. and *Aspergillus* section *Fumigati* between sampling methods. Kruskal–Wallis test.

	Sampling Method	Ranks		Test Statistics ^a,b^		
		N	Mean Rank	Kruskal–Wallis H	df	*p*
Fungal Contamination (CFUs)	Millipore	33	107.21	115.897	5	0.000 *
	Andersen	112	77.39			
	Filter	30	204.05			
	EDC	34	165.87			
	Settled dust	12	112.67			
	Others	15	176.47			
	Total	236				
*Aspergillus* sp. (CFUs)	Millipore	33	104.05	149.849	5	0.000 *
	Andersen	112	73.04			
	Settled dust Filters	30	208.37			
	EDC	34	178.04			
	Settled dust	12	97.13			
	Others	15	192.17			
	Total	236				
*Aspergillus* section *Fumigati*	Millipore	33	106.64	6.787	5	0.237
	Andersen	112	119.06			
	Filter	30	125.27			
	EDC	34	125.35			
	Settled dust	12	99.50			
	Others	15	126.53			
	Total	236				

^a^ Kruskal–Wallis Test. ^b^ Grouping Variable: Sampling Method. * Statistically significant differences at the 5% significance level. * EDC—Electrostatic dust collector; CFUs—Colony forming units.

**Table 6:** Comparison of Fungal contamination, *Aspergillus* sp. and *Aspergillus* section *Fumigati* between media. Kruskal–Wallis test.

	Media	Ranks		Test Statistics ^a,b^		
		N	Mean Rank	Kruskal–Wallis H	df	*p*
Fungal contamination (CFUs)	MEA	71	121.16	20.273	3	0.000 *
	DG18	140	107.38			
	SDA	16	179.69			
	ITR+VOR	9	161.67			
	Total	236				
*Aspergillus* sp. (CFUs)	MEA	71	102.49	27.499	3	0.000 *
	DG18	140	114.99			
	SDA	16	181.16			
	ITR+VOR	9	188.06			
	Total	236				
*Aspergillus* section *Fumigati*	MEA	71	124.52	39.411	3	0.000 *
	DG18	140	108.86			
	SDA	16	131.38			
	ITR+VOR	9	198.06			
	Total	236				

^a^ Kruskal–Wallis test. ^b^ Grouping variable: sampling method. * Statistically significant differences at a 5% significance level. * CFU—Colony forming units; MEA—Malt Extract Agar; DG18—Dichloran–Glycerol Agar; SDA-Sabouraud Dextrose Agar; ITR—Itraconazole; VOR—Voriconazole; POS—Posaconazole.

**Table 7:** Comparison of fungal contamination, *Aspergillus* sp. and *Aspergillus* section *Fumigati* between FFHs. Kruskal–Wallis test.

	FFH	Ranks		Test Statistics ^a,b^		
		N	Mean Rank	Kruskal–Wallis H	df	*p*
Fungal contamination (CFUs)	1	31	115.48	22.200	10	0.014 *
	2	23	108.41			
	3	20	139.53			
	4	7	103.14			
	5	40	78.63			
	6	27	128.44			
	7	13	140.00			
	8	26	137.04			
	9	14	136.14			
	10	22	122.66			
	11	13	136.88			
	Total	236				
*Aspergillus* sp. (CFUs)	1	31	115.90	22.227	10	0.014 *
	2	23	116.57			
	3	20	140.30			
	4	7	127.07			
	5	40	86.88			
	6	27	136.17			
	7	13	168.92			
	8	26	117.31			
	9	14	132.29			
	10	22	99.95			
	11	13	119.08			
	Total	236				
*Aspergillus* section *Fumigati*	1	31	112.00	22.481	10	0.013 *
	2	23	109.20			
	3	20	119.83			
	4	7	99.50			
	5	40	117.89			
	6	27	144.22			
	7	13	137.35			
	8	26	130.62			
	9	14	108.00			
	10	22	104.23			
	11	13	99.50			
	Total	236				

^a^ Kruskal–Wallis Test. ^b^ Grouping Variable: Sampling Method. * Statistically significant differences at the 5% significance level. * CFUs—Colony forming units.

**Table 8:** Relationship between fungal contamination, *Aspergillus* sp. and *Aspergillus* section *Fumigati*. Spearman’s correlation coefficient.

	*Aspergillus* Section *Fumigati*	Fungal Contamination (CFUs)
*Aspergillus* sp. (CFUs)	0.137 *	0.705 **
*Aspergillus* section *Fumigati*		0.130 *

* Correlation is significant at the 0.05 level (2-tailed). ** Correlation is significant at the 0.01 level (two tailed). * CFUs-Colony forming units.

## Data Availability

Through the UIDB/05608/2020 and UIDP/05608/2020.

## Supplementary Materials

The following are available online at https://www.mdpi.com/article/10.3390/microorganisms9102112/s1, Table S1. Samples distribution per sampling method in each FFH. Table S2. Formulas applied for the calculation of CFU. M^−3^/m^−2^ * CFU.m^−2^.day^−1^. Table S3. *Aspergillus* section *Fumigati* distribution in each FFH per sampling method. Table S4. *Aspergillus* section *Fumigati* prevalence in positive samples from FFH in MEA and DG18. Table S5. Identification and detection of the *Aspergillus* section *Fumigati* in the assessed FFH.
