# Supplementary material for: Aspergillus Section Fumigati in Firefighter Headquarters
Source: Microorganisms. 2021 Oct 7;9(10):2112. doi: 10.3390/microorganisms9102112 (PMC8541501; doi:10.3390/microorganisms9102112)
Supplement: Supplementary file 1 [file microorganisms-09-02112-s001.zip › microorganisms-1378941-supplementary.pdf]

Table S1. – Samples distribution per sampling method in each FFH

Sampling method	FFH1	FFH2	FFH3	FFH4	FFH5	FFH6	FFH7	FFH8	FFH9	FFH10	FFH11	TOTAL
Andersen	52	32	36	28	36	36	24	32	28	32	24	360
Millipore	60	36	40	32	36	40	28	36	32	32	28	400
EDC*	13	8	6	6	9	7	6	7	7	7	6	82
Cleaning Cloths	7	2	3	2	1	2	1	2	2	1	2	25
Mops	3	1	1	2	1	1	1	1	1	1	1	14
Badges	17	4	5	6	5	5	5	5	5	5	5	67
Swabs	13	8	9	7	9	9	6	8	7	8	6	90
Filters	13	8	9	7	9	9	6	8	7	8	6	90
Settled dust filters	1	1	1	1	1	1	1	1	1	1	1	11

*EDC - Electrostatic dust collector

Table S2. – Formulas applied for the calculation of CFU. m^{-3} / m^{-2} *CFU.m^{-2}.day^{-1}

Sampling method	Formula applied
Air sampling through impaction method (Andersen and Millipore devices)	$CFU.m^{-3} = \frac{x}{250} 1000$
Surface swabs	$CFU.m^{-2} = \frac{x}{0.0001}$
EDC	$CFU.m^{-2}.day$ =(1 x/(3.14*0.003))/days of sampling
Filters/mops/Cleaning cloths/ firefighter's´ badges	$CFU.m^{-2} = 0.1 \frac{x}{2*10^{-4}}$
Settled dust	$CFU.g^{-1}$

Table S3. – *Aspergillus* section *Fumigati* distribution in each FFH per sampling method

FFH	*Fumigati* positive samples in each sampling method		Section *Fumigati* frequency
2	Andersen	1	0.03
5	Andersen	4	0.11
6	Andersen	4	0.11
7	Andersen	2	0.08
8	Andersen	4	0.13
10	Andersen	1	0.03
8	Millipore	1	0.03
9	Millipore	1	0.03
3	Swabs	1	0.11
3	Filters	1	0.11
8	Filters	1	0.13
5	EDC*	1	0.11

11	EDC*	1	0.17

*EDC - Electrostatic dust collector

Table S4. – *Aspergillus* section *Fumigati* prevalence in positive samples from FFH in MEA and DG18

FFH	Sample	MEA			DG18		
		Section *Fumigati*	*Aspergillus* sp.	Section *Fumigati* %	Section *Fumigati*	*Aspergillus* sp.	Section *Fumigati* %
		CFU. m^{-3} / m^{-2} *CFU.m^{-2}.day^{-1}			CFU. m^{-3} / m^{-2} *CFU.m^{-2}.day^{-1}		
2	Andersen	3.93	486.00	0.81	0.00	1040.44	-
5	Andersen	15.70	486.00	3.23	3.93	1040.44	0.38
6	Andersen	94.23	486.00	19.39	0.00	1040.44	-
7	Andersen	0.00	486.00	-	149.19	1040.44	14.34
8	Andersen	3.93	486.00	0.81	23.56	1040.44	2.26
10	Andersen	3.93	486.00	0.81	0.00	1040.44	-
8	Millipore	20.00	158.16	12.65	0.00	393.00	-
9	Millipore	106.16	158.16	67.12	0.00	393.00	-
3	Swabs	0.00	10000.00	-	30000.00	100000.00	30.00
3	Filters	0.00	128500.00	-	23500.00	52505.00	44.76
8	Filters	500.00	128500.00	0.39	0.00	52505.00	-
5	EDC*	106.16	743.10	14.29	0.00	4638.13	-
11	EDC*	106.16	743.10	14.29	0.00	4638.13	-

* EDC - Electrostatic dust collector

Table S5. - Identification and detection of the *Aspergillus* section *Fumigati* in the assessed FFH

Matrice	Local	CFU.m^{-2} ǀ CFU.m^{-2}. day^{-1} (MEA/DG18)	C_q
Uniform names	FFH9	0/0	24.55
		0/0	25.19
		0/0	25.78
	FFH11	0/0	33.90
Filters	FFH1	0/0	37.65
		0/0	34.86
		0/0	37.64
	FFH2	0/0	34.66
		0/0	35.29
	FFH3	0/0	27.23
		0/0	25.71
		0/0	27.33
		0/0	26.31
		0/0	25.93
		0/0	26.84
		0/0	26.43
		0/0	26.00
		0/23500	28.56
	FFH5	0/0	35.78
		0/0	37.89
		0/0	14.77

		0/0	38.30
		0/0	38.38
		0/0	35.46
		0/0	35.09
		0/0	37.09
		0/0	36.02
	FFH6	0/0	35.92
		0/0	34.49
		0/0	35.57
		0/0	34.84
		0/0	33.67
		0/0	35.96
	FFH7	0/0	36.46
		0/0	38.39
		0/0	34.78
		0/0	36.33
		500/0	37.61
	FFH8	0/0	37.45
		0/0	38.03
		0/0	38.58
		0/0	37.89
		0/0	38.95
		0/0	32.49
		0/0	33.88
		0/0	33.03
	FFH9	0/0	33.29
		0/0	33.17
		0/0	33.14
		0/0	32.98
		0/0	35.45
		0/0	37.47
		0/0	34.27
	FFH10	0/0	35.98
		0/0	33.71
		0/0	35.24
		0/0	33.07
		0/0	36.47
		0/0	34.59
		0/0	35.26
	FFH11	0/0	35.70
		0/0	34.30
		0/0	32.77
		0/0	31.72
Mops	FFH1	0/0	38.09
		0/0	35.49
	FFH3	0/0	34.88
		0/0	37.31
	FFH5	0/0	16.28
	FFH6	0/0	15.69
	FFH10	0/0	34.72
Cleaning cloths	FFH2	0/0	35.42
	FFH4	0/0	36.13
	FFH3	0/0	32.93
		0/0	34.79
	FFH5	0/0	33.77
	FFH6	0/0	32.61
		0/0	31.99

	FFH7	0/0	36.45
	FFH8	0/0	32.28
	FFH9	0/0	30.21
		0/0	33.08
	FFH10	0/0	33.84
	FFH11	0/0	33.35
		0/0	33.83
EDC	FFH1	0/0	36.55
		0/0	36.09
		0/0	34.94
		0/0	17.31
		0/0	36.63
	FFH2	0/0	36.71
		0/0	35.72
		0/0	36.03
		0/0	35.59
	FFH3	0/0	33.96
		0/0	34.62
		0/0	34.42
		0/0	35.69
	FFH4	0/0	33.85
		0/0	34.28
		0/0	38.56
		0/0	35.45
	FFH5	0/0	34.98
		106.16/0	34.16
		0/0	35.81
		0/0	34.12
		0/0	35.99
	FFH6	0/0	35.63
		0/0	35.37
		0/0	36.91
		0/0	33.50
		0/0	35.17
		0/0	34.76
		0/0	35.92
		0/0	36.38
	FFH7	0/0	37.54
		0/0	32.63
		0/0	35.77
		0/0	35.34
		0/0	33.74
		0/0	35.33
	FFH8	0/0	35.80
		0/0	34.00
		0/0	33.92
		0/0	33.35
		0/0	32.50
		0/0	34.07
		0/0	33.95
	FFH9	0/0	33.17
		0/0	33.80
		0/0	37.29
		0/0	35.15
		0/0	26.74
	FFH10	0/0	39.36
		0/0	35.43

	0/0	36.38
	0/0	35.07
	0/0	34.85
	0/0	35.26
	0/0	33.52
	0/0	35.58
	0/0	36.58
	0/0	32.16
FFH11	0/0	34.32
	0/0	36.77
	0/0	33.76
